# The Clinical Application of Dynamic Contrast-Enhanced MRI in Canine Masses of Mesenchymal and Epithelial Origin: A Preliminary Case Series

**DOI:** 10.3390/vetsci11110539

**Published:** 2024-11-04

**Authors:** Chang-Hyeon Cho, Jaehwan Kim, Kidong Eom

**Affiliations:** Department of Veterinary Medical Imaging, College of Veterinary Medicine, Konkuk University, Seoul 05029, Republic of Korea; chcho150@konkuk.ac.kr

**Keywords:** DCE-MRI, K^trans^, k_ep_, V_e_, Vp, soft tissue sarcoma, cholesteatoma, squamous cell carcinoma

## Abstract

Evaluating masses of mesenchymal and epithelial origin accurately using computed tomography (CT) has several limitations in dogs. This study aimed to present dynamic contrast-enhanced magnetic resonance imaging (DCE-MRI) parameters to aid in improving the diagnostic accuracy for masses of mesenchymal and epithelial origin in veterinary medicine. Four dogs diagnosed with benign and malignant soft tissue sarcoma (STS), cholesteatoma, or squamous cell carcinoma underwent CT, conventional MRI, and DCE-MRI. K^trans^ is a quantitative DCE-MRI parameter representing vascular permeability and tissue perfusion and is related to the potential for malignancy. Hemangiopericytomas (Grade II, STS) showed a higher K^trans^ than normal muscle tissue and myxosarcoma (Grade I, STS). Squamous cell carcinoma (a malignant epithelial tumor) also showed a higher K^trans^ than normal muscle tissue and cholesteatoma (a mass originating from keratinized squamous epithelium). These results suggest that higher K^trans^ values may indicate a greater likelihood that a lesion is more malignant. In conclusion, K^trans^ might be useful as a biomarker for evaluating the malignancy of a mass and as an indicator of lesion characteristics in dogs.

## Simple Summary

Masses of mesenchymal and epithelial origin are common in dogs. In humans, dynamic contrast-enhanced magnetic resonance imaging (DCE-MRI) is used for disease diagnosis, treatment response evaluation, and treatment planning. However, only a few studies of DCE-MRI have been reported in veterinary medicine. Therefore, this study aimed to introduce DCE-MRI parameters for masses originating from mesenchymal and epithelial tissues in dogs.

## 1. Introduction

Masses originating from mesenchymal and epithelial tissues are common lesions in dogs. Soft tissue sarcoma (STS) is a collective term for classifying various mesenchymal tumors and accounts for approximately 15% of skin and subcutaneous tumors in dogs [1]. Among them, hemangiopericytomas, also known as solitary fibrous tumors, are a slow-growing STS originating from perivascular cells, occurring in various parts of the body in both humans and dogs, with dogs typically developing them in the skin and subcutaneous tissues of the limbs, especially in large breed, middle-aged, and older dogs [2,3,4]. Myxosarcoma, originating from fibroblastic cells, is also common in middle-aged and older dogs, typically affecting the trunk and limbs and sometimes synovial joints and organs [5,6]. Epithelial masses are common in canine skin, with skin tumors accounting for 26–43% of biopsied masses, 20–40% of which are malignant [1]. Squamous cell carcinoma (SCC) originates from squamous cells and commonly affects the oral cavity and skin in dogs, typically appearing between 8.3 and 9.1 years of age [7]. Cholesteatomas are benign cystic masses in the middle ear, often arising from chronic otitis media and can cause structural damage if they grow progressively [8,9].

Imaging is essential for diagnosing, staging, and treatment planning in oncology. Magnetic resonance imaging (MRI) provides detailed information on masses, helping detect recurrence, predict tumor grade, and plan treatment [10,11,12,13,14]. While commonly used in human medicine, MRI is less accessible in veterinary medicine due to the need for anesthesia, which requires a longer time than CT [15]. As a result, studies on MRI for mesenchymal and epithelial masses are limited in veterinary medicine. 

Contrast enhancement in MRI and CT is important for oncology imaging but occurs in both benign and non-tumoral conditions, limiting its diagnostic accuracy [16,17,18,19]. To overcome this, dynamic contrast-enhanced MRI (DCE-MRI) was developed, which assesses vascular permeability and angiogenesis in tumors. A malignant mass requires neovascularization for growth once it reaches about 1 mm in diameter [20]. These newly formed blood vessels are incomplete, with unstable structures, leading to higher vascular permeability. This increased permeability allows contrast agents and large proteins to leak into the extracellular–extravascular space (EES) [20,21]. DCE-MRI analyzes the movement of contrast agents using pharmacokinetic analysis to assess tissue perfusion and vascular permeability. Quantitative and qualitative parameters were used to analyze masses by DCE-MRI. Various models are available for analyzing quantitative parameters; the extended Tofts model is the most widely used in oncology. In the extended Tofts model, four quantitative parameters are obtained: (1) K^trans^ (the volume transfer constant from blood plasma to EES), (2) k_ep_ (the rate constant from EES to plasma), (3) V_e_ (volume of EES per unit volume of tissue), and (4) Vp (the fractional plasma volume). These parameters are associated with vascular permeability and angiogenesis. K^trans^ is the parameter used most commonly for vascular permeability and is combined with blood flow, permeability, and the capillary surface area. It can differentiate between a malignant and benign mass, evaluate mass type and tumor grade, detect mass recurrence, and assess the response to radiation therapy and chemotherapy [14,22,23]. V_e_*,* Vp, and k_ep_ represent the volume of contrast agent accumulation in the EES, the volume of contrast agent in the plasma, and the reflux rate of the contrast agent from the EES to the plasma, respectively. The time-intensity curve (TIC) is a qualitative DCE-MRI parameter representing the change in signal intensity over time. Analysis of this curve pattern can help assess the malignancy of a mass [21,24,25]. 

Using these parameters, DCE-MRI is used for various diseases, including those affecting the brain, breast, musculoskeletal system, and other diseases [26]. DCE-MRI can help distinguish between benign and malignant soft tissue mass and assess the response to radiotherapy and chemotherapy [14,22,24,25]. Few studies have been conducted on the application of DCE-MRI [27,28,29] in veterinary medicine compared to human medicine. Therefore, this case study aimed to introduce DCE-MRI parameters and findings for masses of mesenchymal and epithelial origin in dogs. 

## 2. Material and Methods

### 2.1. Study Design

This case study included cases in which DCE-MRI was performed on masses of epithelial or mesenchymal origin, confirmed through histopathological diagnosis. We excluded cases in which DCE-MRI was performed without histopathological diagnosis and cases involving masses of epithelial or mesenchymal origin for which DCE-MRI was not performed. The study included four dogs diagnosed with soft tissue tumors, cholesteatoma, and SCC through histopathological examination at the Konkuk Veterinary Teaching Hospital between 2022 and 2024. All tissue specimens were collected via whole mass excision or incisional biopsy performed by veterinary surgeons. Veterinary pathologists performed the histopathological diagnoses and interpretations. Informed consent was obtained from the owners for all patients who underwent CT, MRI, and DCE-MRI. The patient data, including breed, age, sex, weight, imaging modality, and lesion location, are summarized in Table 1.

### 2.2. MRI and CT Examination Protocol

All patients underwent 1.5T MRI (Signa Hdxt, GE Medical Systems, Milwaukee, WI, USA), including conventional MRI sequences and DCE-MRI sequences, and 160-slice CT (Aquilion, Canon Medical Corporation, Tochigi, Japan) that included pre-contrast and post-contrast phases under general anesthesia with ventilation. Patient 1 was positioned in lateral recumbency and scanned using a flexed MRI coil. Patients 2, 3, and 4 were positioned in prone recumbency and scanned using a flexed MRI coil. Conventional MRI data were acquired using the following sequences: T2-weighted (T2W), T2W fluid-attenuation inversion recovery (FLAIR), diffusion-weighted imaging (DWI), Apparent diffusion coefficient (ADC) and pre-contrast and post-contrast phase of the T1-weighted (T1W) sequence or 3D T1W fast spoiled gradient echo sequences. 

DCE-MRI was performed with reference to the Quantitative Imaging Biomarkers Alliance (QIBA) and human studies [30]. Baseline T1 mapping data were acquired with variable flip angles (*α* = 5°, 10°, 15°, 20°, 25°). For the DCE-MRI scan, all patients were administrated a 0.1 mmol/kg gadoteric acid (0.5 mmol/mL Clariscan; GE Healthcare AS; Oslo, Norway) contrast agent bolus at a 2.0 mL/s velocity, followed by 12 mL 0.9% normal saline at the same velocity using a dual MRI contrast injector (Sonic shot 7, Nemoto Kyorindo Co., Ltd.; Tokyo, Japan) via an IV catheter in the cephalic vein. The DCE-MRI parameters are summarized in Table 2. All DCE-MRI data included eight phases of baseline images before administering the contrast agent. During the CT scan, Patient 1 was positioned in supine recumbency, and Patients 2, 3, and 4 were positioned in prone recumbency. The CT data were acquired under the following conditions: slice thickness of 1 mm; matrix size of 512 × 512; helical pitch of 0.81; rotation time of 0.75 s; kVp of 120; and mAs of 112.

For contrast-enhanced CT imaging, all patients were administered 2 mL/kg of a non-ionic contrast agent (300 mg/mL Omnipaque; GE Healthcare Co., Ltd., Shanghai, China) via an IV catheter in the cephalic vein. CT images were acquired during the pre-contrast and post-contrast phases (110 s after contrast injection).

### 2.3. DCE-MRI Data Analysis

**Figure 1:**
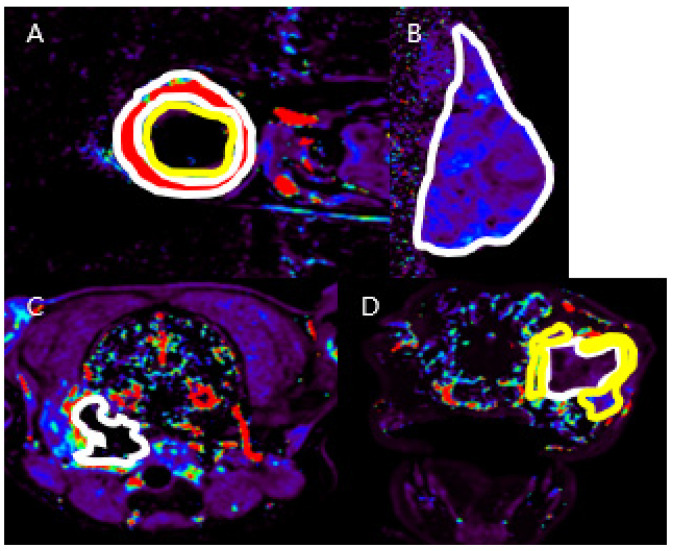
Regions of interest (ROIs) in the DCE-MRI K^trans^ maps. The ROI of the peripheral mass region is outlined by white circles, and the ROI of the central mass region is outlined in yellow in Case 1 (**A**). The ROI of the mass region is outlined in white in Case 2 (**B**) and 3 (**C**). In Case 4 (**D**), the ROI for the peripheral area of the mass is indicated in yellow, while the ROI for the center area of the mass is outlined in white.

DCE-MRI data were analyzed using commercial software (Olea Sphere v3.0, Olea Medical, La Ciotat, France) with the region of interest (ROI) of arterial input function in adjacent large arteries [21]. The quantitative parameters were obtained by post-processing DCE-MRI data using the extended Tofts model: K^trans^, V_e_, Vp, k_ep_. The DCE parameter values were measured by manually drawing ROIs on the K^trans^ map, excluding the hemorrhage and blood vessel areas (Figure 1). Each measurement was performed five times by the same observer and was conducted by three experienced veterinary radiologists.

## 3. Results

### 3.1. Case 1

A 13-year-old castrated male Yorkshire terrier presented with an exophytic mass on the neck. The mass had grown rapidly for 3 months before the visit; no other symptoms were observed. Other laboratory tests did not detect any abnormalities, including a complete blood cell count (CBC), full serum biochemical profile, and thoracic radiography with abdominal ultrasonography. An MRI scan on the neck mass was performed using T2W, T2W FLAIR, DWI, pre-contrast T1W fat saturation, post-contrast T1W fat saturation, and DCE-MRI sequences following a CT scan.

**Figure 2:**
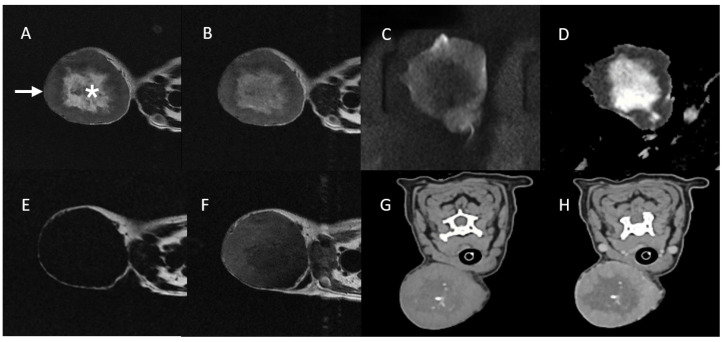
MRI and CT images of Case 1. The central mass region (asterisk) showed heterogeneous T2 hyperintensity (**A**) and T2W FLAIR hyperintensity (**B**), with a low DWI signal (**C**) and a high ADC value (**D**) on MRI. These findings suggest that the central mass region (asterisk) has undergone necrotic changes. The peripheral mass region (arrow) shows a low-to-intermediate T2W hyperintensity (**A**) and T2W FLAIR hyperintensity (**B**) compared to adjacent normal muscle with slightly high DWI signal (**C**) and a low ADC value (**D**). In addition, the mass exhibited hypointensity on pre-contrast T1W fat saturation (**E**), with enhancement of the peripheral mass region on both post-contrast T1W fat saturation (**F**) and post-contrast CT (**H**). Several mineralizations were identified in the central mass region on pre-contrast CT (**G**). These mineralizations in the central mass region on pre-contrast CT (**G**) are suspected to be dystrophic calcification resulting from necrotic changes.

MRI images showed heterogeneous hyperintensity on T2W and T2W FLAIR with a low DWI signal and a high ADC value in the central mass region. The peripheral mass region showed low-to-intermediate hyperintensity on T2W compared to normal muscle, with a slightly high DWI signal and a low ADC value. Additionally, the mass exhibited hypointensity on pre-contrast T1W fat saturation, with enhancement of the peripheral mass region on both post-contrast T1W fat saturation and post-contrast CT. Multifocal mineralizations observed in the central mass region on CT corresponded to the areas of hypointensity seen in the central mass region on T2W image. In addition, the average HU (Hounsfield unit) value of the central mass region, excluding the areas of mineralization, was 20–23 on pre-contrast CT. No metastasis or invasion was identified by MRI or CT (Figure 2). 

**Figure 3:**
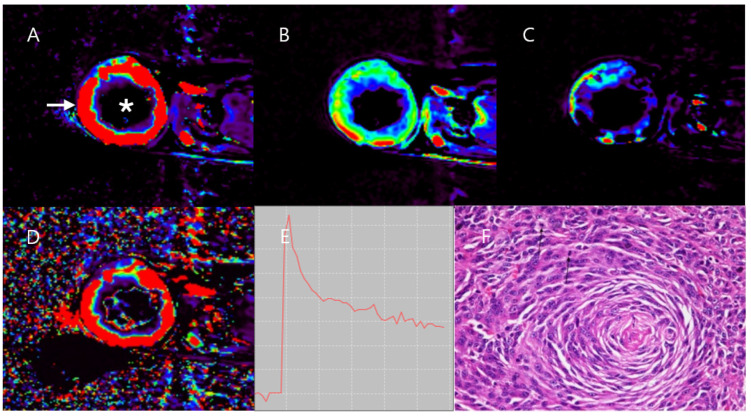
DCE-MRI image and histology image of Case 1. High K^trans^ (**A**), V_e_ (**B**), V_p_ (**C**), and k_ep_ (**D**) in the peripheral mass region are found. In contrast, the central mass region (asterisk) shows much lower K^trans^ (**A**), V_e_ (**B**), V_p_ (**C**), and k_ep_ (**D**) than the peripheral mass region (arrow). TIC (**E**) in the peripheral mass region shows fast wash-in and wash-out patterns. A histopathological image (**F**), stained with hematoxylin and eosin (H&E) and observed at 400× magnification shows hypertrophic nuclei and anisocytosis in mesenchymal mass cells. The histopathological result indicates a diagnosis of grade II hemangiopericytoma.

DCE-MRI data showed higher K^trans^, k_ep_, V_e_, and V_p_ values in the peripheral mass region compared to the central mass region and the adjacent normal muscle region. The K^trans^ and V_e_ values in the central mass region were extremely low. The TIC of the peripheral mass region showed a fast wash-in and wash-out pattern; the TIC of the central mass region showed a plateau pattern (Figure 3). The mass size was measured to be 57.1 × 52.5 × 45.5 mm (length × width × height). After an MRI and CT scan, the patient underwent complete resection surgery with adequate margins. 

A histopathological result revealed a tumorigenic proliferation of cells of mesenchymal origin. The mass exhibited a whorl growth pattern around the blood vessels. Necrosis was observed in approximately 30–40% of the mass, and fibrosis of the stroma was noted. Mass cells proliferating around blood vessels displayed spindle shapes and were densely packed. The nuclei of the mass cells were thickened, ranging from round to oval in shape, and they exhibited anisokaryosis. The mitotic count was 7 per 10 high-power fields. The mass was diagnosed as grade II hemangiopericytoma. During the 2-year follow-up period, no signs of recurrence were observed in the patient who remains alive. 

### 3.2. Case 2

**Figure 4:**
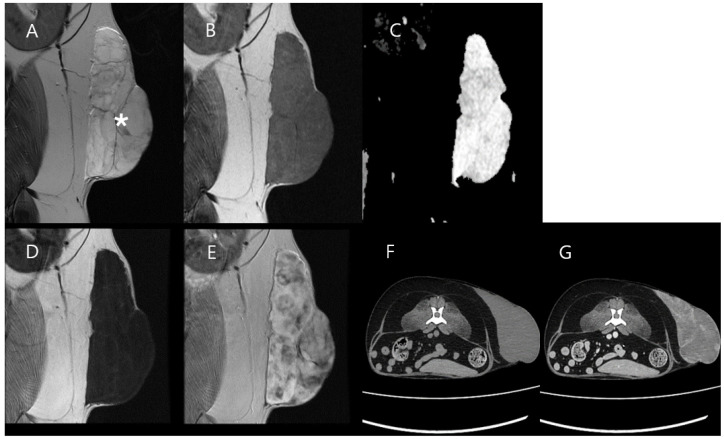
MRI and CT images of Case 2. The mass (asterisk) showed heterogeneous T2W hyperintensity (**A**) and T2W FLAIR hypointensity (**B**) with a high ADC value (**C**). These results suggest that the mass contains myxoid material. Marked hypointensity on pre-contrast T1W (**D**) and heterogeneous enhancement on post-contrast T1W (**E**) are observed on MRI. Low attenuation is identified in pre-contrast CT (**F**), and a slight enhancement is identified on post-contrast CT (**G**).

A 12-year-old castrated male Spitz patient presented with a large mass in the left flank. The mass grew progressively over approximately 7 months. Laboratory tests, including CBC, full serum biochemical profile, and whole-body radiography with abdominal ultrasonography, revealed no abnormalities. An MRI scan was performed on the left flank mass using T2W, T2W FLAIR, DWI, pre-contrast T1W, post-contrast T1W, and DCE-MRI sequences. MRI images showed heterogeneous T2W hyperintensity and T2W FLAIR hypointensity with a high ADC value in the mass. The mass appeared markedly hypointense on pre-contrast T1W and exhibited a heterogeneous enhancement pattern on post-contrast T1W. No obvious finding of metastasis or invasion of the adjacent normal structures was identified on MRI or CT (Figure 4). 

**Figure 5:**
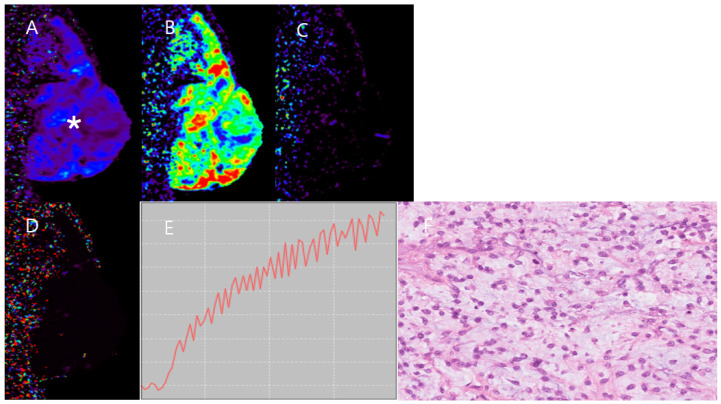
DCE-MRI image and histology image of Case 2. Slightly higher K^trans^ (**A**) and V_e_ (**B**) and slightly lower V_p_ (**C**) and low k_ep_ (**D**) are observed in the mass (asterisk) compared to the adjacent normal muscle. TIC (**E**) in the mass shows a progressive pattern. A histopathological image (**F**), stained with hematoxylin and eosin (H&E) and observed at 400× magnification, shows an abundant myxoid matrix rich in mucopolysaccharides in mesenchymal cells. The histopathological result indicates a diagnosis of grade I myxosarcoma.

DCE-MRI data showed that K^trans^ and V_e_ were slightly higher than in the adjacent normal muscle. The k_ep_ and Vp values were lower for the mass than for normal muscle. The TIC of the mass showed a progressive pattern (Figure 5). The mass size was measured to be 114.3 × 59.8 × 113.1 mm (length × width × height). After an MRI and CT scan, the patient underwent a complete resection surgery with adequate margins.

A biopsy revealed polygonal and spindle-shaped cells containing a moderate amount of eosinophilic to lightly basophilic cytoplasm. Neoplastic cells had round to oval nuclei with a granular chromatin pattern and single large prominent nucleoli embedded in a light-basophilic myxomatous stroma. The mass was diagnosed as grade I myxosarcoma based on the histopathological result. After surgery, no signs of recurrence or metastasis were observed during the 2-year follow-up period; the patient remains alive.

### 3.3. Case 3

**Figure 6:**
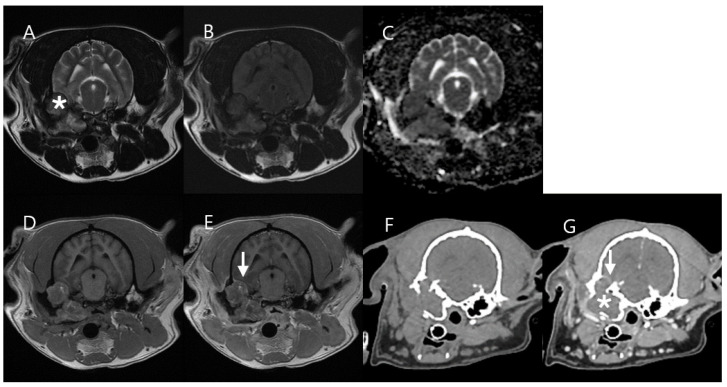
MRI and CT images of Case 3. The mass (asterisk) shows heterogeneous T2W hyperintensity (**A**) and T2W FLAIR hyperintensity (**B**) with a slightly high ADC value (**C**). Additionally, the mass (asterisk) exhibited slight hypointensity on pre-contrast T1W (**D**) and peripheral enhancement around the bulla wall on post-contrast T1W images (**E**). Osteolysis of the right tympanic bone and temporal bone are identified in pre-contrast CT (**F**). Meningeal enhancement in the area (arrow) compressed by the mass is observed on post-contrast T1W images (**E**) and post-contrast CT (**G**).

A 6-year-old sprayed female Jindo mix presented with sudden, intermittent creamy discharge from the right ear for 1 year without any warning symptoms or pain response. The patient had a history of extended chronic otitis externa in both ears and previously received a few months of treatment for cholesteatoma in the right middle ear. Laboratory tests, including CBC, full serum biochemical profile, and whole-body radiography with abdominal ultrasound, revealed no specific findings in the other body parts. An MRI scan was performed on the head with T2W, T2W FLAIR, DWI, pre-contrast T1W, post-contrast T1W, and DCE-MRI sequences after a CT scan. The mass exhibited heterogeneous T2W hyperintensity and T2W FLAIR hyperintensity with a slightly high ADC value. In addition, slight hypointensity on pre-contrast T1W and peripheral enhancement around the bulla wall was identified in the mass on post-contrast T1W images. CT showed bone destruction in the petrous part of the right temporal bone and right tympanic bone, leading to a mass compressing of the meninges of the right temporal lobe, as confirmed by both MRI and CT (Figure 6). 

**Figure 7:**
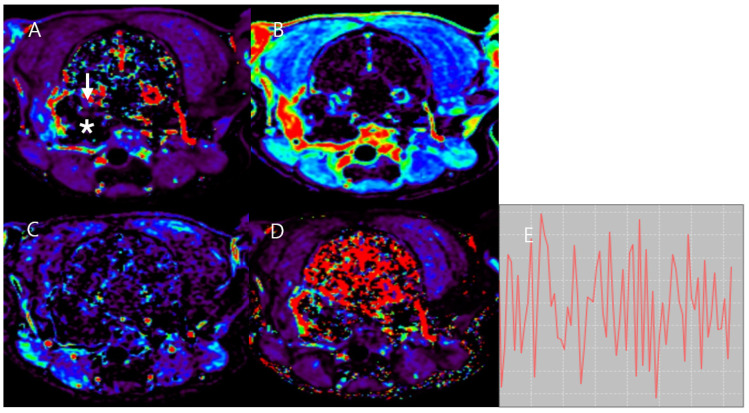
DCE-MRI in Case 3. Slightly elevated K^trans^ (**A**), V_e_ (**B**), V_p_ (**C**), and k_ep_ (**D**) are found in the mass (asterisk). K^trans^ values (**A**) are significantly increased in the peripheral region around the bulla (arrow) and temporal meninges compressed by the mass. The time-intensity curve (**E**) of the mass shows a plateau pattern. This mass was diagnosed as a cholesteatoma through histopathology.

DCE-MRI data showed slightly higher K^trans^ and V_e_ values in the mass region compared to the contralateral bulla region. Higher K^trans^ and V_e_ values were found in the peripheral region around the bulla compared to the mass. The TIC of mass showed a plateau pattern (Figure 7). The mass size was measured to be 26.8 × 23.5 × 30.1 mm (length × width × height). After an MRI and CT scan, the patient underwent total ear canal ablation and lateral bulla osteotomy surgery for a mass in the right middle ear. Histological evaluation of the mass removed by surgery was performed by a veterinary pathologist. A histopathologic result showed that the mass comprised a fibrovascular core surrounded by keratinizing stratified squamous epithelium. The epithelium was irregularly, moderately hyperplastic, and it was coated with abundant lamellar keratin. In one of the extremities, there was a large, ulcerated area, which was infiltrated by numerous viable and degenerate neutrophils, and fewer macrophages and lymphocytes mixed to fibrin and cellular debris. The cells infiltrated the superficial stroma, which was expanded by fibrosis. Deeper in this area, increased numbers of fibroblasts formed bundles parallel to the ulcerated surface, separated by irregularly spaced perpendicular blood vessels lined by plump endothelium (granulation tissue). Therefore, the mass was diagnosed as a cholesteatoma. One month later, the mass recurred in the same area, and a second surgery was performed. Histopathological examination requested after the second surgery also confirmed a diagnosis of cholesteatoma. Since the second surgery, the patient has survived without pain or other symptoms, and no recurrence has been observed. 

### 3.4. Case 4

**Figure 8:**
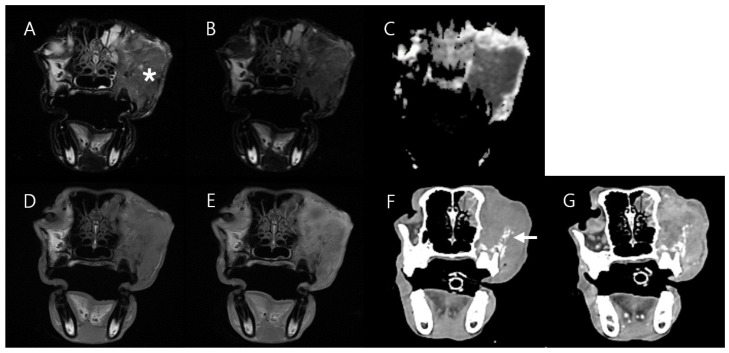
MRI and CT images of Case 4. Intermediate hyperintensity of mass (asterisk) is observed on T2W (**A**) and T2W FLAIR (**B**) images compared to normal muscle. A low ADC value (**C**) is identified in the mass (asterisk), with T1W isointensity (**D**) and heterogeneous enhancement (**E**) on MRI. Multifocal osteolytic lesions (arrow) in the maxilla are found on pre-contrast CT (**F**), and heterogeneous enhancement is observed on post-contrast CT (**G**).

A 9-year-old female Belgian Malinois was diagnosed with a large mass in the left facial region. The affected area swelled rapidly over approximately 3 months, and displacement of the left globe was noted. In addition, pus was observed in the mass on the left side of the face. Laboratory tests showed elevated monocyte and neutrophil levels in CBC. An MRI scan of the left facial region was performed with T2W, T2W FLAIR, DWI, pre-contrast T1W, post-contrast T1W, and DCE-MRI sequences after a CT scan. MRI showed an ill-defined, hyperintense area on T2W with a low ADC value. The mass was isointense on T1W with heterogeneous contrast enhancement. Mass invasion was detected in adjacent structures, including the oral cavity, left maxilla, bilateral nasal cavity, and left frontal sinus, with multifocal osteolytic lesions in the left maxilla and left nasal cavity (Figure 8). 

**Figure 9:**
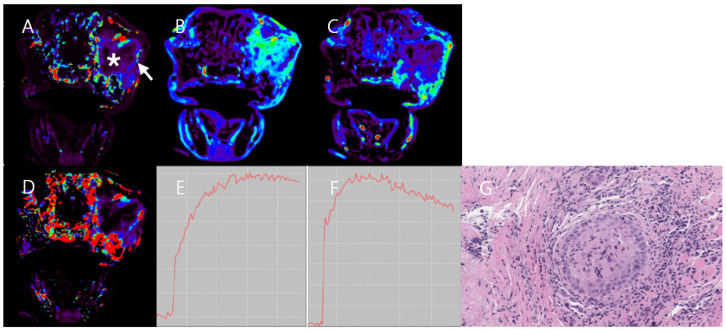
DCE-MRI image and histology image of Case 4. Higher K^trans^ (**A**), V_p_ (**C**), and k_ep_ (**D**) are found in the peripheral mass region (arrow) compared to the central mass region (asterisk) and adjacent normal muscle. The central mass region (asterisk) shows higher K^trans^ (**A**), V_e_ (**B**), V_p_ (**C**), and k_ep_ (**D**) compared to the adjacent normal muscle, and slightly higher V_e_ (**B**) compared to the peripheral mass region (arrow). TIC (**E**) in the central mass region shows a progressive pattern; TIC (**F**) in the peripheral mass region shows a rapid wash-in and slow wash-out pattern. A histopathological image (**G**), stained with hematoxylin and eosin (H&E) and observed at 400× magnification, shows lobules of varying sizes composed of squamous epithelial cells in the dermis. The histopathologic result indicates a diagnosis of SCC in situ based on the imaging findings and the patient’s clinical signs, the mass was ultimately concluded to be SCC by a veterinary radiologist, veterinary pathologist, and veterinary surgeon.

DCE-MRI data showed higher K^trans^, V_p_, and k_ep_ in the peripheral mass region compared to the central mass region and adjacent normal muscle. The TIC of the mass showed a rapid wash-in and slow wash-out pattern in the peripheral region and a progressive pattern in the central region of the mass (Figure 9). The mass size was measured to be 95.8 × 32.4 × 54.3 mm (length × width × height). Histopathology showed lobules of varying sizes composed of squamous epithelium. Neoplastic cells did not invade the basement membrane; no mitotic cells were observed. The mass was diagnosed as SCC in situ based on the histopathologic findings from the incisional biopsy. However, an incisional biopsy specimen is generally considered less accurate than a surgical excision specimen. In light of the imaging findings and the patient’s clinical presentation, the mass was ultimately concluded to be SCC by the veterinary radiologist, veterinary pathologist, and veterinary surgeon.

Surgery could not be performed because of the ill-defined margins of the mass and a clear invasion of the surrounding structures. The patient was scheduled to undergo radiotherapy; however, subsequent contact with the owner was lost.

The DCE-MRI parameter values for masses of mesenchymal and epithelial origin in this study are listed in Table 3 and Table 4, respectively.

## 4. Discussion

This study observed differences in the DCE-MRI parameters across the four canine cases, including two high-grade tumors (Case 1 and Case 4), one low-grade tumor (Case 2), and a benign mass (Case 3). In summary, the high-grade tumors (Case 1 and Case 4) showed significantly higher K^trans^ values compared to the low-grade tumor (Case 2) and benign mass (Case 3), which reflects increased vascular permeability associated with malignancy. A low-grade tumor (Case 2) demonstrated slightly higher K^trans^ values than a benign mass (Case 3), consistent with the expectation of reduced angiogenesis and vascular permeability in benign lesions. These findings align with previous studies indicating a correlation between malignancy and DCE-MRI parameters, such as K^trans^, where higher values are typically associated with more aggressive tumors [14,23,31,32]. Notably, the K^trans^ values observed in Cases 1, 2, and 4 were higher compared to those typically seen in human medicine. This is likely due to the faster blood flow in small animals, as K^trans^ is influenced by blood flow velocity [20].

Case 1 was diagnosed with grade II hemangiopericytoma, considered a malignant and vascularized tumor [33,34]. MRI and DCE-MRI showed necrosis and dystrophic calcification in the central mass region [35,36,37,38,39,40,41], while the peripheral mass region exhibited high cellularity and neovascularization. DCE-MRI revealed elevated K^trans^, k_ep_, V_e_, and Vp in the peripheral region, indicating high vascular permeability due to tumor cell proliferation, as confirmed by histopathology. In contrast, the central mass region had very low K^trans^ and V_e_, likely due to extensive necrosis and the loss of blood vessels in the core of the mass, which minimized the movement of the contrast agent from the blood plasma to the EES in the necrotic region [42,43]. This was confirmed by histopathology. The DCE-MRI parameters and TIC patterns in Case 1 were similar to those observed in human medicine [31,32]. 

Case 2 was diagnosed as grade I myxosarcoma. DCE-MRI showed a slightly higher K^trans^ values compared to normal muscle but significantly lower than in Case 1 (grade II hemangiopericytoma). The lower K^trans^ reflects the tumor’s low malignancy, consistent with human studies showing that low-grade STSs have lower K^trans^ values [32]. The TIC pattern in Case 2 was similar to benign myxoid tumors [25], but comprehensive MRI and K^trans^ evaluations indicated low-grade malignancy. Since TIC is a qualitative parameter with lower diagnostic accuracy than quantitative parameters, it cannot be solely relied upon, making the comprehensive assessment crucial for an accurate diagnosis. 

Case 3 was diagnosed with cholesteatoma, a benign cystic mass with low K^trans^ values due to minimal angiogenesis [9]. However, the peripheral region around the bulla showed higher K^trans^ due to inflammation. The TIC pattern displayed a plateau, differing from human benign lesions [24]. Unlike quantitative parameters such as K^trans^, TIC is qualitative, making it less accurate for distinguishing between benign and malignant lesions. Therefore, a TIC pattern alone cannot reliably indicate a high likelihood of malignancy. MRI and DCE-MRI findings suggested a benign mass, which was confirmed by biopsy. 

Case 4 was diagnosed with SCC based on imaging, clinical presentation, and histopathology. DCE-MRI showed significantly higher K^trans^ values in the mass compared to normal muscle and Case 3. However, the peripheral mass region had higher K^trans^ values than the central mass region, because many malignant masses undergo rapid growth and expansion, often leading to an inability to meet the metabolic demands required for such growth. Consequently, the tumor center receives less blood supply, causing hypoxia, low pH, low glucose, and high lactate levels, which lead to necrosis [43]. As a result, the central region shows lower K^trans^ values compared to the more vascularized peripheral region [44]. The TIC pattern in the peripheral mass region matched human studies, while the central mass region’s pattern differed, possibly due to hypoxia-induced ischemia [21,24]. 

In this study, DCE-MRI was conducted following QIBA guidelines to improve reproducibility and repeatability. These guidelines recommend acquiring a baseline T1 map and using an automatic MRI injector with high temporal and spatial resolution. Balancing temporal resolution, spatial resolution, and signal-to-noise ratio (SNR) is crucial for ensuring high-quality DCE-MRI data [30,45]. 

This study has a limitation. Due to the small sample size, diagnostic criteria for DCE-MRI parameters according to disease type were not established. However, this study was designed as a preliminary case study with the primary aim of identifying early trends in DCE-MRI parameters for masses of mesenchymal and epithelial origin. Although these trends are exploratory, they may provide a valuable starting point for future research, particularly in veterinary applications. Additionally, similar trends have been reported in clinical studies involving DCE-MRI in human patients, lending further support to these preliminary findings. To confirm the findings of this study, further research with a larger sample size is necessary, and follow-up studies are expected to provide more definitive validation of the findings presented in this paper. 

DCE-MRI provides hemodynamic information about the mass microenvironment. This information can help improve diagnostic accuracy, assess tumor grade, predict prognosis, and assess treatment response [14,22,23,32]. In addition, recent studies have shown that DCE-MRI parameters are associated with mass heterogeneity, including hypoxia, histological features, and molecular markers including isocitrate dehydrogenase mutation, 1p19q co-deletion, O^6^-methylguanine-DNA-methyltransferase promoter methylation, and epidermal growth factor receptor levels [23]. However, quantitative DCE-MRI parameters commonly used in human medicine, including K^trans^, have not been reported in veterinary medicine [27,28,29]. Therefore, further studies on applying DCE-MRI in veterinary clinical practice would help inform disease diagnosis, treatment response evaluation, and treatment planning.

## 5. Conclusions

Since contrast enhancement occurs in multiple conditions, including masses, inflammation, vascular diseases, and trauma, making an accurate diagnosis solely with conventional CT and MRI may be limited. Therefore, DCE-MRI has been clinically applied to complement conventional CT and MRI in human medicine, aiding in more accurate diagnoses and treatment planning. Similarly, in veterinary medicine, DCE-MRI can provide additional information about lesion characteristics, complementing conventional CT and MRI, and may help improve diagnostic accuracy. 

This case study reported DCE-MRI findings and quantitative parameters for masses of mesenchymal and epithelial origin. The K^trans^ values were higher in high- grade STS and SCC cases than in low-grade STS and cholesteatoma cases. These results suggest that higher K^trans^ values may be associated with a greater likelihood that the lesion is more malignant with more active neovascularization and higher vascular permeability in veterinary cases, similar to findings in human studies. Therefore, K^trans^ might be useful as a biomarker for evaluating the malignancy of a mass and as an indicator of lesion characteristics in dogs.

## Figures and Tables

**Table 1:** Summary of patient’s data.

Case	Breed	Age (year)	Sex	Weight (kg)	Imaging Modality	Lesion Location
1	Yorkshire Terrier	13	Castrated male	4.6	Conventional MRI, DCE-MRI, CT	Neck (Subcutaneous)
2	Spitz	12	Castrated male	11.3	Conventional MRI, DCE-MRI, CT	Lt. flank (Subcutaneous)
3	Jindo mix	6	Sprayed female	12.3	Conventional MRI, DCE-MRI, CT	Rt. middle ear
4	Belgian Malinois	9	Female	17.2	Conventional MRI, DCE-MRI, CT	Lt. facial region

Abbreviation: MRI, magnetic resonance imaging; DCE-MRI, dynamic contrast-enhanced magnetic resonance imaging; CT, computed tomography; Lt.; left; Rt.; right.

**Table 2:** Summary of DCE-MRI parameters.

Case	Pre-T1 Map	Flip Angle	TR (ms)	TE (ms)	Voxel Size (mm^3)^	Temporal Resolution (s)
1	X *	35°	7	2	1.6 × 1.6 × 10	5
2	O	25°	4	1	1.2 × 1.2 × 4	5
3	O	15°	5	2	1.2 × 1.2 × 4	5
4	O	15°	4	1	1.2 × 1.2 × 4	5

* Because the anesthesia time of Patient 1 was extended, the pre-T1 map could not be obtained. Abbreviation: DCE, dynamic contrast enhancement; MRI, magnetic resonance imaging; Pre-T1 map, pre-contrast T1 mapping; TR, repetition time; TE, echo time; voxel size, volume element size.

**Table 3:** The DCE-MRI parameter values for masses of mesenchymal origin in this study.

	Case 1 *		Case 2 *	
Mass Type	Soft Tissue Sarcoma (Hemangiopericytoma, Grade II)	Normal Region (Adjacent Muscle)	Soft Tissue Sarcoma (Myxosarcoma, Grade I)	Normal Region (Adjacent Muscle)
K^trans^ (min^−1^), mean ± SD	1.393 ± 0.026 (Peripheral mass region) 0.013 ± 0.079 (Central mass region)	0.089 ± 0.042	0.176 ± 0.064	0.059 ± 0.141
k_ep_ (min^−1^) mean ± SD	5.137 ± 0.643 (Peripheral mass region) 0.992 ± 1.409 (Central mass region)	0.381 ± 0.118	0.305 ± 0.108	0.699 ± 0.883
V_e_ mean ± SD	0.499 ± 0.107 (Peripheral mass region) 0.014 ± 0.043 (Central mass region)	0.239 ± 0.089	0.585 ± 0.209	0.088 ± 0.115
Vp mean ± SD	0.241 ± 0.099 (Peripheral mass region) 0.011 ± 0.00006 (Central mass region)	0.001 ± 0.00006	0.003 ± 0.00006	0.019 ± 0.045
TIC Type	Fast wash-in, Fast wash-out (Peripheral mass region) Plateau (Central mass region)	Progressive	Progressive	Plateau

* Histopathological examination was performed by veterinary pathologists following surgical excision. Abbreviations: DCE, dynamic contrast enhancement; MRI, magnetic resonance imaging; TIC, time-intensity curve; SD, standard deviation; K^trans^, volume transfer constant; k_ep_, rate constant; V_e_, extracellular extravascular space volume fraction; Vp, plasma volume fraction; min^−1^, per minute.

**Table 4:** The DCE-MRI parameter values for masses of epithelial origin in this study.

	Case 3 *		Case 4 **	
Mass Type	Cholesteatoma	Normal Region (Contralateral Bulla)	Squamous Cell Carcinoma	Normal Region (Contralateral Muscle)
K^trans^ (min^−1^), mean ± SD	0.043 ± 0.1	0	0.24 ± 0.136 (Central mass region) 0.667 ± 0.143 (Peripheral mass region)	0.058 ± 0.074
k_ep_ (min^−1^) mean ± SD	1.812 ± 2.318	0	0.83 ± 0.561 (Central mass region) 2.866 ± 0.568 (Peripheral mass region)	0.57 ± 1.911
V_e_ mean ± SD	0.023 ± 0.052	0	0.296 ± 0.064 (Central mass region) 0.236 ± 0.038 (Peripheral mass region)	0.144 ± 0.041
Vp mean ± SD	0.001 ± 0.014	0	0.103 ± 0.046 (Central mass region) 0.188 ± 0.048 (Peripheral mass region)	0.065 ± 0.055
TIC Type	Plateau	Absent	Progressive (Central mass region) Rapid wash-in, Slow wash-out (Peripheral mass region)	Progressive

* Histopathological examination was performed by veterinary pathologists following surgical excision. ** Histopathological examination was performed by veterinary pathologists following incisional biopsy. Abbreviations: DCE, dynamic contrast enhancement; MRI, magnetic resonance imaging; TIC, time-intensity curve; SD, standard deviation; K^trans^, volume transfer constant; k_ep_, rate constant; V_e_, extracellular extravascular space volume fraction; Vp, plasma volume fraction; min^−1^, per minute.

## Data Availability

Data from this study are available from the corresponding author upon request.
